# Is the association between blood pressure and cognition in the oldest-old modified by physical, vascular or brain pathology markers? The EMIF-AD 90 + Study

**DOI:** 10.1186/s12877-023-04440-w

**Published:** 2023-11-11

**Authors:** Nienke Legdeur, Justine E. Moonen, Maryam Badissi, Carole H. Sudre, Wiesje Pelkmans, Mark Forrest Gordon, Frederik Barkhof, Mike Peters, Pieter Jelle Visser, Majon Muller

**Affiliations:** 1 Alzheimer Center Amsterdam, Neurology, Vrije Universiteit Amsterdam, Amsterdam UMC Location VUmc, Amsterdam, The Netherlands; 2 Amsterdam Neuroscience, Neurodegeneration, Amsterdam, The Netherlands; 3 Department of Internal Medicine, Spaarne Gasthuis, Haarlem, The Netherlands; 4 MRC Unit for Lifelong Health and Ageing at UCL, University College London, London, UK; 5 Centre for Medical Image Computing, Department of Computer Science, University College London, London, UK; 6 School of Biomedical Engineering and Imaging Sciences, King’s College London, London, UK; 7 Dementia Research Centre, Institute of Neurology, University College London, London, UK; 8 Barcelonaβeta Brain Research Center (BBRC), Pasqual Maragall Foundation, Barcelona, Spain; 9 Teva Pharmaceuticals, West Chester, PA USA; 10 Department of Radiology & Nuclear Medicine, Amsterdam Neuroscience, Vrije Universiteit Amsterdam, Amsterdam UMC Location VUmc, Amsterdam, The Netherlands; 11 Institutes of Neurology and Healthcare Engineering, University College London, London, UK; 12 Department of Geriatrics, UMC Utrecht, Utrecht, The Netherlands; 13 Department of Psychiatry & Neuropsychology, School for Mental Health and Neuroscience, Maastricht University, Maastricht, The Netherlands; 14 Department of Neurobiology, Care Sciences Division of Neurogeriatrics, Karolinska Institutet, Stockholm, Sweden; 15 Department of Internal-Geriatric Medicine, Amsterdam Cardiovascular Sciences, Vrije Universiteit Amsterdam, Amsterdam UMC Location VUmc, Amsterdam, The Netherlands

**Keywords:** Blood pressure, Cognition, Vulnerability, Oldest-old

## Abstract

**Background:**

Prior studies suggest a changing association between blood pressure (BP) and cognition with aging, however work in the oldest-old has yielded ambiguous results. Potentially, these mixed results can be explained by modifying factors. The aim of this study was to establish whether physical, vascular or brain pathology markers that describe a state of increased vulnerability, affect the association between BP and cognition in the oldest-old. Results may influence clinicians’ decisions regarding the use of antihypertensives in this age group.

**Methods:**

We included 122 individuals (84 without cognitive impairment and 38 with cognitive impairment) from the EMIF-AD 90 + Study (mean age 92.4 years). First, we tested cross-sectional associations of systolic and diastolic BP with a cognitive composite score. Second, we tested whether these associations were modified by physical markers (waist circumference, muscle mass, gait speed and handgrip strength), vascular markers (history of cardiac disease, carotid intima media thickness as a proxy for atherosclerosis and carotid distensibility coefficient as a proxy for arterial stiffness) or brain pathology markers (white matter hyperintensities and cortical thickness).

**Results:**

In the total sample, there was no association between BP and cognition, however, waist circumference modified this association (*p*-value for interaction with systolic BP: 0.03, with diastolic BP: 0.01). In individuals with a high waist circumference, higher systolic and diastolic BP tended to be associated with worse cognition, while in individuals with a low waist circumference, higher systolic BP was associated with better cognition. The others physical, vascular and brain pathology markers did not modify the association between BP and cognition.

**Conclusions:**

When examining various markers for physical, vascular and brain vulnerability, only waist circumference affected the association between BP and cognition. This warrants further research to evaluate whether waist circumference may be a marker in clinical practice influencing the use of antihypertensives in the oldest-old.

**Supplementary Information:**

The online version contains supplementary material available at 10.1186/s12877-023-04440-w.

## Background

High blood pressure (BP) during midlife has been related to cognitive decline [1, 2], but in late-life this association seems to change [3–5]. Some studies found no association between BP and cognition in older individuals, while others even observed a reverse relation [6–9]. The underlying pathophysiological mechanisms that may explain the altered association of BP with cognition in late-life and the variability in results within the older individuals are largely unknown.

**Fig. 1:**
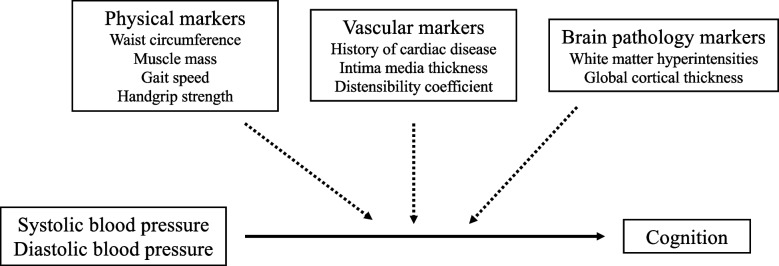
Illustration of the investigated interaction analyses

It has been hypothesized that the level of frailty determines whether an older individual is more susceptible to the negative consequences of a low BP [10–12]. Frailty refers to a state of increased vulnerability to dependency and morbidity as a consequence of deterioration of various physiological systems [13]. In literature, frailty is mostly referred to as *physical* frailty of which the definition by Fried et al. is the most familiar one [14]. In the present paper, we will extend this definition of frailty by also assessing vulnerability of other physiological systems, namely the vascular and nervous system [13]. Individuals with a positive history of cardiac disease, atherosclerosis or an increased arterial stiffness are known to be at greater risk for increased dependency and morbidity [15, 16]. These measures might therefore be used as markers for vulnerability of the vascular system. Brain pathologies, such as white matter hyperintensities and cortical atrophy, have been related to morbidity and mortality and may represent a state of increased brain vulnerability [17, 18]. Last, next to the physical markers that are part of the frailty definition by Fried et al. (gait speed and handgrip strength), additional physical markers, such as waist circumference and muscle mass, have been related to an increased frailty risk and may also be considered as markers of physical vulnerability [19, 20]. All these physical, vascular and brain pathology markers may modify the association of BP with cognition (Fig. 1). Since the presence of these markers is inextricably related to age, their modifying effect may be mostly relevant in the oldest-old (individuals aged 90 years and older). Furthermore, identifying which individuals are more susceptible to the consequences of low or high BP is important, as it may impact the use of antihypertensive medications. Therefore, this study aims to establish the association between BP and cognition in the oldest-old and explore whether physical, vascular and brain pathology markers that relate to increased vulnerability modify this association (Fig. 1). We hypothesize that the oldest-old who are more vulnerable based on one of the described physical, vascular or brain pathology markers, are more susceptible to cognitive deterioration in the presence of a low BP [10–12].

## Methods

### Study population

Individuals were included from the European Medical Information Framework for Alzheimer’s disease (EMIF-AD) 90 + Study. The aim of the EMIF-AD 90 + Study was to establish protective factors for cognitive impairment in the oldest-old. Therefore, the design of the study was a case–control study including both cognitively normal and cognitively impaired individuals aged 90 years and older [21]. Inclusion criteria for cognitively normal individuals were a global Clinical Dementia Rating (CDR) score of 0 and a score ≥ 26 points on the Mini-Mental State Examination (MMSE) [22]. Three individuals with an MMSE < 26 points were included in the cognitively normal group, as they were determined to be cognitively normal after extensive cognitive testing. Inclusion criteria for individuals with cognitive impairment were a diagnosis of amnestic mild cognitive impairment (aMCI) [23] or a diagnosis of probable or possible Alzheimer’s disease (AD) [24] and a global CDR score ≥ 0.5. Exclusion criteria were physical inability to undergo the procedures, visual or hearing impairment interfering with neuropsychological testing, severe depression and comorbidities or medications that could impair cognition, as judged by the investigator. Individuals were recruited from June 2016 to July 2018 via advertisement, outreach to general practitioners (GPs), and the 100-plus Study [25]. The Medical Ethical Committee of the Amsterdam UMC approved this study. All individuals provided written informed consent before participating in the study. The EMIF-AD 90 + Study was registered in the Nederlands Trial Register NTR5867 on 20 May 2016.

### Clinical characteristics

Data about the medical history, medication use, and education were collected through structured interview, in combination with information provided by the study partner (husband, wife, family member, friend, or caregiver) if available, GP, and/or medical specialist. The presence of hypertension, diabetes mellitus, and dyslipidemia was based on a positive medical history and/or medication use.

### Blood pressure

Blood pressure (BP) was measured three times in lying position using a sphygmomanometer. The measurement took place at the hospital and participants had to lie down for five minutes before the measurement took place. In the analyses, the mean of the three systolic and three diastolic BP measures was used. For three individuals, BP was based on one BP measurement and for one individual on two BP measurements. Seven individuals did not have a sphygmomanometer BP measurement. For these individuals, the first measurement of a continuous BP measure was used, which was assessed with a digital photoplethysmogram on the right middle finger (Nexfin®, BMEYE, Amsterdam, The Netherlands). The use of seven BP measurements with the Nexfin was found acceptable as in individuals who had both measurements, there was a small (< 10 mmHg) difference between mean systolic and diastolic BP measured with a sphygmomanometer (153/78 mmHg) and mean systolic and diastolic BP measured with the Nexfin (145/76 mmHg).

### Cognitive composite score

Cognitive tests were administered by a trained neuropsychologist. We computed a cognitive composite score to reduce the number of outcome measures, and thereby the chance of a type 1 error. This was done by calculating z-scores (with higher values representing better scores) of fourteen cognitive tests (Table 1), adding these z-scores together per cognitive domain (memory, processing speed and executive functioning) and dividing the sum by the number of cognitive tests per cognitive domain. An average score of these three domain scores was calculated and after that scaled to compose a cognitive composite z-score. There was no time limit for the assessment of the TMT A and B. However, some individuals could not perform the TMT B due to cognitive problems. Therefore, we assigned maximum scores to the TMT A and B in these individuals to minimize the number of missing values on the TMT B/A ratio. Maximum scores were based on the time 2SD above the study sample mean. To avoid outliers that might influence associations, all scores higher than the maximum score were limited to the maximum score.

**Table 1:** Cognitive tests

	Scoring range	Cognitive domain
CERAD 10-words test [26]		
Immediate recall	0–30	Memory
10-min delayed recall	0–10	Memory
Logical memory test [27]		
Immediate recall	0–23	Memory
20–30-min delayed recall	0–23	Memory
Rey Complex Figure Test delayed copy after 3 min corrected for the immediate copy [28]	0-36^a^	Memory
Visual Association Test [29]	0–12	Memory
Total adjusted errors on the PAL test of the CANTAB [30]	0–70	Memory
Median five-choice reaction time of the CANTAB	N/A	PS
Digit Symbol Substitution Test from the WAIS-R [31]	N/A	PS
Trail Making Test (TMT) A [32]	N/A^b^	PS
Trail Making Test B corrected for A [32]	N/A^b^	EF
Clock Drawing Test [33]	0–14	EF
Letter fluency (1 min per letter, letters D-A-T) [34]	0–93	PS
Digit Span Backward from the WAIS-III [35]	0–14	EF

*CANTAB* computerized Cambridge Neuropsychological Test Automated Battery, *CERAD* Consortium to Establish a Registry for Alzheimer's Disease; *EF* executive functioning, *N/A* not applicable, *PAL* Paired Associate Learning, *PS* processing speed, *WAIS(-R)* Wechsler Adult Intelligence Scale(-Revised). ^a^This is the range for the copies separately; ^b^There was no time limit for the assessment of the TMT A and B, but for some individuals scores were missing due to cognitive problems, therefore we assigned maximum scores based on the time 2SD above the study sample mean (see text for a more extensive explanation)

### Modifying factors

#### Physical markers

The following four physical markers were included: waist circumference, muscle mass, gait speed and handgrip strength. Waist circumference was measured in centimeters at the level of the umbilicus and was used as a measure of central adiposity [36]. We selected waist circumference, as central obesity has a stronger association with various cardiovascular health outcomes than overall weight/BMI (body mass index) [36]. In addition, although fat mass was also determined in the EMIF-AD 90 + Study by a bioelectrical Impedance Analyzer (BIA), we did not use this measurement in the present study. The reason is that fat mass by BIA seems to be less stable and comparable between individuals than waist circumference as it is influenced by various factors, such as sex, age and underlying medical conditions [36].

The BIA (InBody 770 or S10; Biospace Co., Ltd, Seoul, Korea) was used to measure skeletal muscle mass in kilograms [37]. The skeletal muscle mass index (further described as muscle mass) was calculated by dividing skeletal muscle mass by height squared (kg/m^2^).

To determine gait speed, individuals were asked to walk 4 m at their usual speed twice, with or without walking aid. The fastest time was used to calculate their gait speed (m/sec).

Handgrip strength of the dominant hand was measured twice with a hand dynamometer (Jamar hand dynamometer; Sammons Preston, Inc., Bolingbrook, IL., USA). The highest score in kilograms was used in the analyses [38].

#### Vascular markers

Three vascular markers were used: a medical history of cardiac disease, the intima media thickness (as measure of atherosclerosis) and the distensibility coefficient (as measure of arterial stiffness) of the right common carotid artery.

Cardiac disease was defined as a positive medical history of angina pectoris, myocardial infarction, a percutaneous coronary intervention or heart failure. Data about medical history were collected through structured interview and, when necessary due to incompleteness, supplemented by information provided by study partner, GP and/or medical specialist.

To determine intima media thickness and the distensibility coefficient, the right common carotid artery was scanned with ultrasound at 10 mm proximal from the carotid bulb using a 7.5-MHz linear array probe. With the use of ArtLab software, the diameter (D), intima media thickness (IMT) and distension (ΔD) of the carotid artery were assessed [39]. The following formula was used to calculate the distensibility coefficient (DC) [40]:

$$\mathrm{DC }= (2\mathrm{\Delta D x D }+\Delta {\mathrm{D}}^{2}) / (\mathrm{PP x }{\mathrm{D}}^{2})$$

where PP is the brachial pulse pressure, calculated as the systolic minus the diastolic BP. Prior to the analyses, DC values were log transformed because of their skewed distribution.

#### Brain pathology markers

The following two imaging markers for brain pathology were included: global cortical thickness and white matter hyperintensity volume. Brain MRI-scans were performed on a Philips 3 T Achieva scanner and structural three-dimensional (3D) T1-weighted images and 3D sagittal fluid-attenuated inversion recovery (FLAIR) sequences were acquired with isotropic 1 mm resolution [41]. A neuroradiologist visually inspected the MRI-scans for incidental findings.

Global cortical thickness (further referred to as ‘cortical thickness’) was estimated from the 3DT1 MRI using FreeSurfer (v5.3; http://surfer.nmr.mgh.harvard.edu/). Non-brain tissue was removed, followed by transformation to MNI space, segmentation and creation of cortical surface meshes [42]. Next, we calculated global cortical thickness as the weighted average across hemispheres (left cortical thickness relative to left total brain volume, plus right cortical thickness relative to right total brain volume, divided by total brain volume). White matter hyperintensity (WMH) segmentation was performed using an algorithm based on a three-level Gaussian mixture model to model healthy tissues and lesions [43]. Both the FreeSurfer and WMH segmentations were visually inspected and cortical thickness data from five individuals and WMH data from two individuals were excluded due to gross registration and segmentation errors. To correct for head size, WMH was expressed as percentage of total intracranial volume (TIV, which is the sum of grey matter, white matter and cerebrospinal fluid). WMH values were log transformed to correct for its skewed distribution.

### Statistical analyses

First, systolic and diastolic BP were associated with the cognitive composite score using linear regression analyses adjusted for age, sex and years of education. Second, interaction analyses were performed to test whether these associations were modified by the physical, vascular or brain pathology markers (Fig. 1). The modifying factors were, one at a time, added as interaction term with systolic or diastolic BP to the linear regression analyses adjusted for age, sex and years of education. All modifying factors were added as continuous variables, except for the presence of cardiac disease, which was dichotomous.

Results of the interaction analyses were presented with a forest plot using sim_slopes in R [44]. For interpretation purposes, the standardized regression coefficients of the—1SD and + 1SD value of the modifying factor were shown in the forest plot (except for cardiac disease, as this is a dichotomous variable, standardized regression coefficients for present/absent were shown).

As supplemental analyses, the above-mentioned potential modifying factors were also presented for the cognitively normal and impaired individuals separately, and associated with the cognitive composite score using linear regression analyses adjusted for age, sex and years of education, in order to enhance the interpretation of the results of the interaction analyses.

Two sensitivity analyses were performed. First, to explore whether results were driven by the cognitively impaired individuals, the linear regression between BP and cognition and the interaction analyses adjusted for age, sex and years of education were performed in only the cognitively normal individuals. Second, to explore whether results changed due to the seven participants who had a different BP measure method (with the Nexfin instead of a sphygmomanometer), the same analyses were repeated without these seven participants.

The *p*-value threshold for significance was set at 0.05. Statistical analyses were performed in R-Studio version 2022.7.1.554 with R version 4.2.1.

## Results

Characteristics and sample sizes per variable of the 122 individuals included in the EMIF-AD 90 + Study are shown in Table 2. Not every measure could be performed in all individuals due to logistic and individual specific reasons (for example not able to lie down in the MRI-scanner). Individuals were on average 92.4 years old (SD 2.8, IQR: 90.5–93.5 years), 70 (57.4%) were female, their median years of education was 10.0 (IQR 9.0–13.0), 38 (31.1%) individuals were cognitively impaired and 84 (68.9%) individuals had hypertension. The cognitively impaired individuals were slightly younger, had a lower gait speed, handgrip strength (for males) and global cortical thickness than the cognitively normal individuals (Supplementary Table S1).

**Table 2:** Characteristics of the total study sample

	Total	
	N	
Age, y	122	92.4 (2.8)
Sex, female^a^	122	70 (57.4)
Education, y^b^	122	10.0 (9.0–13.0)
MMSE, points	122	27.0 (3.1)
CERAD immediate recall, words	114	15.6 (4.8)
Cognitively impaired^a,c^	122	38 (31.1)
Hypertension^a,d^	122	84 (68.9)
Diabetes Mellitus^a,d^	122	8 (6.6)
Dyslipidemia^a,d^	121	38 (31.4)
Systolic BP, mmHg	109	151.9 (24.3)
Diastolic BP, mmHg	109	78.0 (11.8)
Waist circumference, cm	116	100.1 (11.2)
Muscle mass index, kg/m^2^	95	9.1 (1.0)
Gait speed, m/sec	102	0.8 (0.2)
Handgrip strength females, kg	66	11.5 (4.6)
Handgrip strength males, kg	50	21.3 (7.0)
Cardiac disease^a,e^	121	42 (34.7)
IMT, mm	99	0.7 (0.1)
Distensibility coefficient, 10–3/kPa^b,f^	82	10.7 (3.3–20.3)
WMH volume, % ICV^b,f^	90	1.3 (0.6–2.2)
Global cortical thickness, mm	87	2.2 (0.1)

Values are presented as mean (SD), unless stated otherwise. BP: blood pressure; CERAD: Consortium to Establish a Registry for Alzheimer's Disease; *ICV* intracranial volume, *IMT* intima media thickness, *MMSE* Mini-Mental State Examination; N: sample size per variable in the total study sample; WMH: white matter hyperintensity; y: years. ^a^Presented as number (%); ^b^Presented as median (IQR); ^c^Clinical diagnosis of amnestic mild cognitive impairment or probable/possible Alzheimer’s disease; ^d^Based on medical history and/or medication use; ^e^Positive medical history of angina pectoris, myocardial infarction, a percutaneous coronary intervention or heart failure, ^f^These values are log transformed in the analyses

### Associations with cognition

Systolic BP and diastolic BP were not associated with cognition in the oldest-old (Table 3). In addition, there was no association of waist circumference, a medical history of cardiac disease, IMT or the distensibility coefficient with cognition. However, higher muscle mass, gait speed, handgrip strength and global cortical thickness and lower WMH volumes were associated with better cognition.

**Table 3:** Associations of blood pressure and the physical, vascular and brain pathology markers with cognition^a^

	β^b^(95% CI)
Systolic BP, mmHg	0.03 (-0.09 to 0.16)
Diastolic BP, mmHg	-0.02 (-0.14 to 0.10)
*Physical markers*	
Waist circumference, cm	0.08 (-0.05 to 0.21)
Muscle mass index, kg/m^2^	0.17 (0.01 to 0.34)*
Gait speed, m/sec	0.22 (0.10 to 0.34)*
Handgrip strength, kg	0.33 (0.18 to 0.47)*
*Vascular markers*	
Cardiac disease	0.03 (-0.23 to 5.26)
IMT, mm	0.04 (-0.08 to 0.17)
Distensibility coefficient, 10–3/kPa^c^	0.05 (-0.08 to 0.19)
*Brain pathology markers*	
WMH volume, % ICV^c^	-0.17 (-0.30 to -0.03)*
Global cortical thickness, mm	0.14 (0.00 to 0.28)*

*BP* blood pressure, *CI* confidence interval. ^a^Cognition is represented by a cognitive composite z-score and a higher value represents a better score; ^b^Standardized regression coefficient (except for cardiac disease which is not scaled because it is a dichotomous variable) from linear regression analysis adjusted for age, sex and years of education; ^c^These values are log transformed in the analyses. *P-value < 0.05

### Interaction analyses

**Fig. 2:**
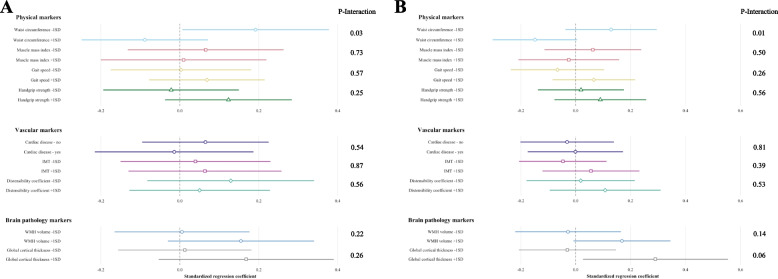
**A** Forest plot of the interaction effects of physical, vascular and brain pathology markers in the association of systolic BP with cognition. BP: blood pressure; IMT: intima media thickness; WMH: white matter hyperintensities, SD: standard deviation. The P-Interactions on the right side of the forest plot are the p-values representing the significance of the interaction term, e.g. systolic BP * waist circumference. In addition, separate analyses associating systolic BP with the cognitive composite z-score were performed for the -1SD or + 1SD (or absence/presence of cardiac disease) values of the physical, vascular and brain pathology markers. The standardized regression coefficients with 95% confidence interval of these analyses are represented by the horizontal lines. Linear regression analyses are adjusted for age, sex and years of education. Values of the distensibility coefficient and WMH are log transformed in the analyses. **B** Forest plot of the interaction effects of physical, vascular and brain pathology markers in the association of diastolic BP with cognition. BP: blood pressure; IMT: intima media thickness; WMH: white matter hyperintensities, SD: standard deviation. The P-Interactions on the right side of the forest plot are the p-values representing the significance of the interaction term, e.g. diastolic BP * waist circumference. In addition, separate analyses associating diastolic BP with the cognitive composite z-score were performed for the -1SD or + 1SD (or absence/presence of cardiac disease) values of the physical, vascular and brain pathology markers. The standardized regression coefficients with 95% confidence interval of these analyses are represented by the horizontal lines. Linear regression analyses are adjusted for age, sex and years of education. Values of the distensibility coefficient and WMH are log transformed in the analyses

Waist circumference modified the association between BP and cognition (p-value for interaction with systolic BP: 0.03, with diastolic BP: 0.01) (Fig. 2A and B). In individuals with a low waist circumference, higher systolic BP was associated with better cognitive functioning, whereas in individuals with a high waist circumference, higher systolic BP tended to be associated with worse cognition (Fig. 2A). For waist circumference and diastolic BP, the same tendency was found: in individuals with a low waist circumference, higher diastolic BP tended to be associated with better cognitive functioning, whereas in individuals with a high waist circumference, higher diastolic BP tended to be associated with worse cognition (Fig. 2B).

There was no modifying effect by the other physical, vascular and brain pathology markers in the association between BP and cognition (Fig. 2).

### Sensitivity analyses

To explore whether results were driven by the cognitively impaired individuals, analyses were repeated in the cognitively normal individuals (N = 84). Similar to the total group, there was no association between BP and cognition in the cognitively normal group: ß (95% CI) for systolic BP: 0.00 (-0.11 to 0.10) and ß (95% CI) for diastolic BP: -0.02 (-0.12 to 0.08). The modifying effect of waist circumference in the association of systolic and diastolic BP with cognition was in the same direction, however, in the cognitively normal individuals this interaction was not significant (p-value for interaction with systolic BP: 0.39; p-value for interaction with diastolic BP: 0.83). In accordance with the interaction analyses in the total group, there were no other significant modifying factors.

To explore whether the seven participants with a different BP measure method (with the Nexfin instead of a sphygmomanometer) had a significant impact on the results, analyses were repeated without these seven participants. These also showed no association between BP and cognition and the results of the interaction analyses were similar.

## Discussion

In this group of oldest-old individuals there was no cross-sectional association of blood pressure (BP) with cognition. However, the association between BP and cognition depended on an individual’s waist circumference, such that in individuals with a low waist circumference, higher systolic BP is preferred for a better cognition. The other physical, vascular and brain pathology markers did not modify the association between BP and cognition.

The absence of an association between BP and cognition (or dementia incidence) in the oldest-old is a replication of the findings published in an earlier study about the EMIF-AD 90 + Study and in line with some of the previous literature [8, 9, 45]. However, there are also studies indicating a lower risk of cognitive impairment, cognitive decline or dementia incidence in the oldest-old with hypertension [6, 46], and a single study indicating a higher risk with very high systolic BP (approximately > 200 mmHg) [47]. Therefore the aim of the present study was to extend these earlier findings to examine whether differences in an individual’s level of vulnerability explain the varying results in these studies. We found that waist circumference modified the association between BP and cognition. High waist circumference, as measure of central adiposity, has been associated with worse cognition when present during midlife, but in later life mixed results have been found [48, 49]. Furthermore, in the Framingham Offspring Study a synergistic adverse effect of hypertension and central adiposity on cognition in individuals aged 40–69 years at baseline was found [48]. This is largely in line with the results of the present study showing a trend towards an adverse effect of high BP on cognition in individuals with a high waist circumference. We extend the findings of the Framingham Offspring Study by showing that in oldest-old individuals with a low waist circumference, high systolic BP is preferred for a better cognition. In individuals aged 65 years and older, larger waist circumference was associated with an increased risk for frailty (defined by a Frailty Index) [19]. However, in the oldest-old, a U-shaped association might be present in which also a low waist circumference is related to increased vulnerability. In these individuals, waist circumference might be an indicator of malnutrition or deterioration of their general health, leading to a higher vulnerability to the negative consequences of a low BP [10]. Furthermore, we did not find a modifying effect by the other markers for physical vulnerability (muscle mass, gait speed and handgrip strength) in the association between BP and cognition. A recent study, conducted in the Brazilian Longitudinal Study of Aging, combined five markers for frailty (similar to the definition by Fried): exhaustion based on a questionnaire, self-reported weight loss, weakness based on handgrip strength, slow gait speed, and low level of physical activity based on a questionnaire [12, 14]. They found that in nonfrail individuals (aged 65 years and older), hypertension was associated with cognitive impairment and in frail individuals, hypertension was related to better cognitive scores. Future studies may extend the findings of the present study by examining whether waist circumference is sufficient to determine which individuals may or may not benefit from antihypertensive treatment with regard to their cognitive functioning, as waist circumference is a much easier and quicker measure to assess than the comprehensive frailty assessment used in the Brazilian Longitudinal Study of Aging.

In line with the physical markers, we hypothesized that markers for vascular vulnerability would lead to a higher susceptibility for cognitive impairment in the presence of a low BP. However, in the oldest-old, we did not find such an interaction. Previous studies conducted in younger individuals, mostly found greater cognitive decline in the presence of more cardiovascular risk factors, which is the opposite from our hypothesis [50, 51]. It might be that in older individuals these two hypotheses interact with each other, in other words, low BP is necessary to slow down the progression of vascular diseases such as atherosclerosis but it also impairs brain perfusion leading to a higher risk of cognitive decline [6]. Furthermore, we found that none of the vascular markers (cardiac disease, IMT and the distensibility coefficient), nor BP itself, was associated with worse cognition in the oldest-old. The interacting pathological processes of different cardiovascular risk factors becomes more complex at higher age. The effect of single parameters might therefore be less distinct then in younger individuals.

The imaging markers to assess brain pathologies (WMH volume and cortical atrophy) also did not modify the association between BP and cognition. We hypothesized that the presence of these brain pathologies is related to a higher state of brain vulnerability and that in these individuals, higher BP should be necessary to preserve cognition [17]. The absence or presence of such an interaction has, to the best of our knowledge, not been described before, which might indicate that other studies also did not find such an interaction. High BP during midlife is a risk factor for both cortical atrophy and WMH [52], and earlier studies mostly indicate a mediating effect of cortical atrophy or WMH in the association between BP and cognition [53]. However, we did not find an association between BP and cognition in the oldest-old and therefore mediation analyses did not apply. We did replicate in the present study that WMH and cortical atrophy were associated with worse cognition in the oldest-old [9]. However, oldest-old individuals with extensive WMH or cortical atrophy were not more vulnerable for cognitive impairments in the presence of a low BP than individuals with more preserved brains, and therefore BP management should not be adjusted based on the level of brain vulnerability in the oldest-old.

The current study has both strengths and limitations. The oldest-old population included in the current study, is unique in how extensively the individuals are phenotyped [21]. Therefore, we were able to include divergent physical, vascular and brain pathology markers to assess vulnerability. Also, as the prevalence of these markers obviously increases with age, the oldest-old are extremely suitable for studying the effect of vulnerability on the association of BP with cognition. There are a few limitations with regard to our methods. First, this is a cross-sectional study and no information about BP during midlife was available. A longer duration of hypertension is associated with lower cognitive scores, and the duration of hypertension might be another modifying factor in the association between BP and cognition [54]. Adding duration of hypertension to our analyses, would have increased insight regarding this hypothesis. Second, we decided not to correct for multiple testing as conventional methods such as Bonferroni correction are overly conservative when various outcomes tests are correlated [55]. Third, the limited sample size in the present study confined its statistical power. For these reasons, our results are exploratory and should be interpreted with caution. Future studies are therefore necessary to replicate the current findings. Fourth, as in all studies conducted in the oldest-old, survival bias and selection bias towards healthier oldest-old individuals might influence the results and potentially explain why certain associations found in younger populations, could not be replicated in the present study. Fifth, in the present study we did not perform a frailty measure, such as the one by Fried. Last, with sensitivity analyses we showed that the modifying effect by waist circumference was in the same direction when only considering the cognitively normal individuals, and that the use of a different BP measure method for seven individuals did not change our results.

## Conclusion

In conclusion, when considering various physical, vascular and brain pathology markers to assess vulnerability, the present study indicated that only waist circumference is a modifier of the association between BP and cognition in the oldest-old. This interaction suggests that waist circumference might be considered in clinical practice when deciding on antihypertensive therapy, such that in individuals with a low waist circumference, higher BP should be targeted to preserve cognition. However, future studies are necessary to confirm the present findings and further elucidate the value of waist circumference in clinical practice.

### Supplementary Information

**Additional file 1: Table S1.** Characteristics of cognitively normal and impaired individuals.

## Data Availability

The datasets generated and analyzed during the current study are available through the EMIF-AD portal, or are available from the corresponding author on reasonable request.

## Abbreviations

AD: Alzheimer’s disease
aMCI: Amnestic mild cognitive impairment
BMI: Body mass index
BP: Blood pressure
CANTAB: Computerized Cambridge Neuropsychological Test Automated Battery
CDR: Clinical Dementia Rating
CERAD: Consortium to Establish a Registry for Alzheimer's Disease
DC: Distensibility coefficient
EMIF-AD: European Medical Information Framework for Alzheimer’s disease
FLAIR: Fluid-attenuated inversion recovery
GP: General practitioners
HbA1c: Hemoglobin A1c
ICV: Intracranial volume
IMT: Intima media thickness
MMSE: Mini-Mental State Examination
PAL: Paired Associate Learning
SD: Standard deviation
TIV: Total intracranial volume
TMT: Trail Making Test
WAIS-R: Wechsler Adult Intelligence Scale-Revised
WMH: White matter hyperintensities
