# Supplementary material for: Is the association between blood pressure and cognition in the oldest-old modified by physical, vascular or brain pathology markers? The EMIF-AD 90 + Study
Source: BMC Geriatr. 2023 Nov 11;23:733. doi: 10.1186/s12877-023-04440-w (PMC10640754; doi:10.1186/s12877-023-04440-w)
Supplement: Supplementary file 1 — Additional file 1: Table S1. Characteristics of cognitively normal and impaired individuals. [file 12877_2023_4440_MOESM1_ESM.docx]

**Supplementary Table S1. Characteristics of cognitively normal and impaired individuals**

|  | CN | CI^a^ | p-value^b^ |
| --- | --- | --- | --- |
| Age, y | 92.8 (2.9) | 91.6 (2.4) | **0.02** |
| Sex, female^c^ | 45 (53.6) | 25 (65.8) | 0.29 |
| Education, y^d^ | 10.0 (9.0-13.0) | 13.0 (9.2-13.0) | 0.32 |
| MMSE, points | 28.5 (1.5) | 23.8 (3.3) | **<0.01** |
| CERAD immediate recall, words | 17.6 (3.8) | 11.2 (3.5) | **<0.01** |
| Hypertension^c,e^ | 58 (69.0) | 26 (68.4) | 1.00 |
| Diabetes Mellitus^c,e^ | 5 (6.0) | 3 (7.9) | 0.70 |
| Dyslipidemia^c,e^ | 24 (28.9) | 14 (36.8) | 0.51 |
| Systolic BP, mmHg | 152 (26.2) | 151.6 (19.6) | 0.93 |
| Diastolic BP, mmHg | 77.9 (11.9) | 78.4 (11.8) | 0.85 |
| Waist circumference, cm | 100.6 (10.8) | 99 (12.0) | 0.51 |
| Muscle mass index, kg/m^2^ | 9.2 (1.0) | 8.8 (1.0) | 0.09 |
| Gait speed, m/sec | 0.8 (0.3) | 0.7 (0.2) | **<0.01** |
| Handgrip strength females, kg | 12.3 (4.0) | 9.9 (5.4) | 0.08 |
| Handgrip strength males, kg | 22.5 (6.7) | 17.5 (6.8) | **0.04** |
| Cardiac disease^c,f^ | 29 (34.9) | 13 (34.2) | 1.00 |
| IMT, mm | 0.7 (0.2) | 0.7 (0.1) | 0.89 |
| Distensibility coefficient, ﻿10-3/kPa^d,g^ | 11.4 (3.6-20.5) | 8.9 (2.9-16.4) | 0.52 |
| WMH volume, % ICV^d,g^ | 1.3 (0.6-2.1) | 1.6 (0.6-2.8) | 0.26 |
| Global cortical thickness, mm | 2.3 (0.1) | 2.2 (0.1) | **0.02** |

*Note:* Values are presented as mean (SD), unless stated otherwise. BP: blood pressure; CERAD: Consortium to Establish a Registry for Alzheimer's Disease; ICV: intracranial volume; IMT: intima media thickness; MMSE: Mini-Mental State Examination; WMH: white matter hyperintensity; y: years. ^a^Clinical diagnosis of amnestic mild cognitive impairment or probable/possible Alzheimer’s disease; ^b^Significance of the difference between cognitively normal and impaired individuals was tested using t-tests, Mann-Whitney U tests or ﻿Fisher’s exact tests were appropriate; ^c^Presented as number (%); ^d^Presented as median (IQR); ^e^Based on medical history and/or medication use; ^f^Positive medical history of angina pectoris, myocardial infarction, a percutaneous coronary intervention or heart failure, ^g^These values are log transformed in the analyses.
